# A Gene Variant in CERS2 Is Associated with Rate of Increase in Albuminuria in Patients with Diabetes from ONTARGET and TRANSCEND

**DOI:** 10.1371/journal.pone.0106631

**Published:** 2014-09-19

**Authors:** Dov Shiffman, Guillaume Pare, Rainer Oberbauer, Judy Z. Louie, Charles M. Rowland, James J. Devlin, Johannes F. Mann, Matthew J. McQueen

**Affiliations:** 1 Celera, Alameda, CA, United States of America; 2 Population Health Research Institute, Hamilton Health Sciences and McMaster University, Hamilton, Ontario, Canada; 3 Department of Nephrology, KH Elisabethinen, Linz, Austria and Department of Nephrology, Medical University of Vienna, Vienna, Austria; 4 Department of Nephrology and Hypertension, Friedrich Alexander University, Erlangen, Germany; University of Tokushima, Japan

## Abstract

Although albuminuria and subsequent advanced stage chronic kidney disease are common among patients with diabetes, the rate of increase in albuminuria varies among patients. Since genetic variants associated with estimated glomerular filtration rate (eGFR) were identified in cross sectional studies, we asked whether these variants were also associated with rate of increase in albuminuria among patients with diabetes from ONTARGET and TRANSCEND—randomized controlled trials of ramipril, telmisartan, both, or placebo. For 16 genetic variants associated with eGFR at a genome-wide level, we evaluated the association with annual rate of increase in albuminuria estimated from urine albumin:creatinine ratio (uACR). One of the variants (rs267734) was associated with rate of increase in albuminuria. The annual rate of increase in albuminuria among risk homozygotes (69% of the study population) was 11.3% (95%CI; 7.5% to 15.3%), compared with 5.0% (95%CI; 3.3% to 6.8%) for heterozygotes (27% of the population), and 1.7% (95%CI; −1.7% to 5.3%) for non-risk homozygotes (4% of the population); P = 0.0015 for the difference between annual rates in the three genotype groups. These estimates were adjusted for age, sex, ethnicity, and principal component of genetic heterogeneity. Among patients without albuminuria at baseline (uACR<30 mg/g), each risk allele was associated with 50% increased risk of incident albuminuria (OR = 1.50; 95%CI 1.15 to 1.95; P = 0.003) after further adjustment for traditional risk factors including baseline uACR and eGFR. The rs267734 variant is in almost perfect linkage-disequilibrium (r^2^ = 0.94) with rs267738, a single nucleotide polymorphism encoding a glutamic acid to alanine change at position 115 of the ceramide synthase 2 (CERS2) encoded protein. However, it is unknown whether CERS2 function influences albuminuria. In conclusion, we found that rs267734 in CERS2 is associated with rate of increase in albuminuria among patients with diabetes and elevated risk of cardiovascular disease.

## Introduction

Chronic kidney disease (CKD) is a common complication of type 2 diabetes and is becoming an increased burden on the medical system. Half of all patients with end stage renal disease starting dialysis suffer from type 2 diabetes mellitus [1]. In patients with diabetes, CKD frequently manifests as albuminuria prior to measurable decline in kidney function as assessed by estimated glomerular filtration rates (eGFR). It has been estimated that about 30% of patients with type 2 diabetes become either micro- or macro-albuminuric within 10 years of diagnosis [2]. However, the rate of CKD progression in patients with diabetes is modest: on average only 2% to 5% become micro-albuminuric each year [2], [3]. Identification of patients with diabetes who are at high risk for CKD progression could help focus medical resources on preventing their progression to end-stage renal disease. Several risk factors for CKD progression including systolic blood pressure, urinary albumin, plasma creatinine, and ethnicity were identified in UKPDS, an observational study of about 5,000 patients with type 2 diabetes [4].

Genetic analysis of kidney disease has largely focused on cross-sectional and case-control studies. A genome-wide meta-analysis investigated the association between genetic variants and eGFR in about 90,000 individuals from mostly population-based studies. This cross-sectional analysis identified 16 single nucleotide polymorphisms (SNPs) that were associated with eGFR at a genome-wide significance level (P<5×10^−8^) [5]. The association of these 16 SNPs with incident CKD was investigated in about 26,000 individuals in eight population-based studies [6]. Only 6 of these loci were associated with incident CKD after adjustment for baseline eGFR. However, these genetic analyses were conducted in population based studies that were not enriched for patients with diabetes. More recently, these 16 SNPs were investigated in a prospective study of about 3000 patients with type 2 diabetes [7]. After correction for multiple testing, none of these SNPs was associated with incident CKD.

The association of these 16 SNPs with albuminuria was investigated in a large cross-sectional study of individuals with European ancestry [8]. In this study the minor allele of a SNP in the SHROOM3 gene was associated with low albuminuria (i.e., better kidney function), a puzzling finding since this same allele is associated with low levels of eGFR.

In this current study we investigated 16 SNPs, that were reported to be associated with eGFR, among patients with type 2 diabetes from European and non-European ancestry enrolled in two randomized controlled trials of ramipril, telmisartan, both, or placebo (ONTARGET and TRANSCEND). We asked whether these 16 SNPs are also associated with early kidney disease measured by accelerated increase in albuminuria.

## Methods

### Study design

This substudy of ONTARGET (ClinicalTrials.gov Identifier: NCT00153101) and TRANSCEND (ClinicalTrials.gov Identifier: NCT00153101) investigated whether 16 SNPs that were previously shown to be associated with eGFR in a cross sectional analysis are also associated with annual rate of increase in albuminuria among patients with diabetes. The ONTARGET and TRANSCEND studies were previously described [9]–[11]. Briefly, these studies included patients 55 years and older with atherosclerotic vascular disease or diabetes and retinopathy, left ventricular hypertrophy, micro- or macro-albuminuria, or a history of cardiac or vascular disease. Patients in ONTARGET were randomized to ramipril (10 mg/day), telmisartan (80 mg/day), or both. Patients intolerant to angiotensin converting enzyme inhibitors were included in the TRANSCEND study and were randomized to telmisartan (80 mg/day) or placebo. Patients in both studies were followed up for 56 months. This substudy included patients who had prevalent or incident diabetes and was drawn from ∼10,000 patients who provided blood samples for DNA analysis. Reasonable efforts were made to collect blood samples for DNA from all ONTARGET/TRANSCEND patients. However, in some countries funding constraints and other difficulties prevented transfer of samples to the core laboratory in Hamilton, and some individual centers or investigators declined to participate. Additionally, blood samples from ∼2000 patients enrolled in China were not included because authorities in Beijing would not allow export of DNA. Diabetes was defined as fasting glucose>125 mg/dl, 2 hour oral glucose tolerance test>199 mg/dl, use of antidiabetic medication, or new diabetes reported by the physician. Albuminuria was estimated from measurement of uACR from a first morning spot urine sample and eGFR was calculated from serum creatinine measurement using the Modification of Diet in Renal Disease (MDRD) formula. Genotyping was conducted using the Cardio-Metabo Chip from Illumina (San Diego CA) which interrogates about 200,000 SNPs, mostly from regions previously shown to be associated with risk of cardiovascular disease, type 2 diabetes, and related quantitative traits [12]. Quality control was performed according to Fellay et al. [13] Briefly, we excluded samples that had <99% call rate. The call rate was calculated based on a total of 189,632 SNPs (96.4% of all Metabochip SNPs) after exclusion of markers that did not achieve>99% call frequency (n = 6,281), markers that had poor signal clustering [13] (n = 783), or markers that had low signal intensity (n = 29). Genotyping reproducibility for 34 samples that were run in duplicates was>99.99%. Genotypes for SNPs in this study that were not present on the Cardio-Metabo Chip were assessed by allele-specific polymerase chain reaction (PCR) [14]. All the SNPs in this study did not deviate from Hardy-Weinberg equilibrium expectations (P>10^−6^). This substudy of ONTARGET and TRANSCEND was approved by the Hamilton Health Sciences and McMaster University Research Ethics Board. All participants provided a written informed consent.

### Statistical analysis

Participant characteristics at baseline were described by counts and percent, by medians and inter-quartile ratios, or by means and standard deviations (Table 1). Deviation from Hardy-Weinberg equilibrium expectations was assessed by an exact test for bi-allelic markers.

**Table 1:** Baseline characteristics.

	Patients in Genetic Analysis		All Patients	
Baseline Characteristic	ONTARGET (n = 3128)	TRANSCEND (n = 595)	ONTARGET (n = 13094)	TRANSCEND (n = 3255)
Age, years, mean (SD)	66.6 (7.2)	67.8 (7.5)	66.1 (6.9)	66.8 (7.2)
Male, n (%)	2197 (70.2)	309 (51.9)	9205 (70.3)	1772 (54.4)
Systolic blood pressure, mmHg, mean (SD)	142.5 (17.4)	141.1 (17.4)	143.2 (17.1)	142.0 (16.6)
Diastolic blood pressure, mmHg, mean (SD)	81.0 (10.3)	80.6 (9.9)	82.1 (10.3)	81.9 (10.2)
Hypertension, n (%)	1900 (60.7)	348 (58.5)	8279 (63.3)	1982 (61.0)
uACR, mg/g, median (IQR)	6.2 (2.7 to 29.4)	4.7 (2.6 to 12.7)	6.5 (2.7 to 30.5)	5.3 (2.5 to 14.5)
<30 mg/g, n (%)	2241 (71.6)	500 (84.0)	8957 (68.4)	2525 (77.6)
30 to 300 mg/g, n (%)	539 (17.2)	56 (9.4)	2196 (16.8)	387 (11.9)
>300 mg/g, n (%)	201 (6.4)	6 (1.0)	813 (6.2)	61 (1.9)
Missing baseline data, n (%)	147 (4.7)	33 (5.5)	1128 (8.6)	282 (8.7)
eGFR, ml/min/1.73 m2, mean (SD)	74.0 (21.0)	71.5 (20.5)	73.3 (20.7)	71.7 (20.1)
Ever smoked, n (%)	2071 (66.2)	327 (55.0)	8160 (62.4)	1599 (49.3)
Self-reported ethnicity				
European, n (%)	2377 (76.0)	430 (72.3)	9146 (69.9)	1879 (57.7)
Asian, n (%)	419 (13.4)	109 (18.3)	1927 (14.7)	758 (23.3)
Native Latin, n (%)	172 (5.5)	35 (5.9)	1233 (9.4)	444 (13.6)
African, n (%)	160 (5.1)	21 (3.5)	424 (3.2)	70 (2.2)
Treatment group				
Placebo, n (%)	NA	299 (50.3)	NA	1629 (50.0)
Ramipril, n (%)	1031 (33.0)	NA	4346 (33.2)	NA
Telmisartan, n (%)	1067 (34.1)	296 (49.7)	4463 (34.1)	1626 (50.0)
Ramipril and Telmisartan, n (%)	1030 (32.9)	NA	4285 (32.7)	NA
Diabetes				
Incident, n (%)	649 (20.7)	154 (25.9)	2524 (19.3)	790 (24.3)
Prevalent, n (%)	2479 (79.3)	441 (74.1)	10570 (80.7)	2465 (75.7)

SD, standard deviation. IQR, inter-quartile range. NA, not applicable.

For regression models that included SNPs, genotypes were coded as 0, 1, or 2 pre-specified risk alleles using an additive model. Because genetic risk factors can have different magnitude in different ethnic groups, we investigated SNPs in ethnic groups separately. Specifically, SNPs were investigated in 4 ethnic groups: self-reported Europeans, Asians (including self-reported Chinese, Japanese, South Asians, other Asians, and Malaysians), Africans (self-reported Black African and Colored African) and self-reported Native Latin. Self-reported Arabs or Persians (n = 9) and others (n = 118) were excluded from the analysis. EIGENSTRAT software [15] was used to calculate the principal components of genetic variability separately in each of the 4 ethnic groups. The principal components were calculated using genotypes from about 50,000 autosomal SNPs that were selected for each ethnic group from the Cardio-Metabo Chip, such that they were not in linkage disequilibrium with one another (r^2^<0.2 in each ethnic group). Patients who were identical by descent (n = 11), or those for whom the first 3 principal components of genetic variability clearly indicated that their genetic ancestry differed from their self-reported ethnicity (n = 34) were excluded. In the basic model, regression models adjusted for age, sex, and to adjust for population structure, the 10 largest ethnic-specific principal components. Results from the 4 ethnic groups were combined using random effects models [16]. The dependent variables eGFR and uACR were natural-log transformed. A fully adjusted model also included baseline hypertension (systolic blood pressure ≥140 mmHg or diastolic blood pressure ≥90 mmHg), baseline eGFR, baseline uACR, smoking status, study (ONTARGET or TRANSCEND), and treatment group. Associations between SNPs and baseline albuminuria or eGFR were assessed using linear regression models. Associations between SNPs and annual rate of change in albuminuria or change in eGFR were assessed using linear mixed models allowing for patient specific random intercepts and slopes. The fixed effects included in the linear mixed models were, visit (0, 2, or 5 years), SNP (number of risk alleles), an interaction term between visit and SNP and remaining basic model variables. The general model in equation form is:



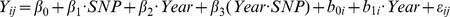
where:


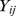
 is the observed measure of urine albumin at Year j for subject i, i = 1, …, M subjects; j = 1, …, n_i_ visits.


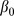
 and 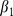
are combined to estimate mean genotype specific population intercepts.


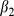
 and 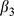
are combined to estimate mean genotype specific population slopes.


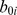
 estimates the deviation from the population intercept for subject i and are assumed independent and Gaussian with mean 0 and variance 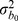.


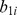
 estimates the deviation from the population slope for subject i and are assumed independent and Gaussian with mean 0 and variance 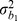.


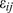
 represents the random deviation of the j^th^ measurement for the i^th^ subject and are assumed independent and Gaussian with mean 0 and variance 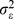.

Additional covariates included in the models allow for additional variation in the population intercepts. The models were fit using the “lme” function found in the R package “nlme” [17]–[19]

A Wald test of the regression coefficient for the interaction term between visit and SNP was used to test whether the rates of change in the measurement over time differed according to the number of risk alleles. Since some mixed models did not converge when baseline uACR was included as a covariate, the fully adjusted models were evaluated using a two-stage linear regression procedure. In the first stage, a linear regression model regressing the measurement as a function of the visit (0, 2 or 5 years) was performed for each patient separately resulting in an estimated slope (rate of change in the measurement per year) for each patient. In the second stage, we used regression models to estimate the effect per risk allele on patient specific slopes. In models that assessed the rate of change in albuminuria, patients with baseline uACR ≥100 mg/g (n = 401) were excluded because this analysis was focused on worsening albuminuria among patients that do not already have substantially elevated levels of urine albumin. Using a similar rationale, models that assessed the rate of eGFR change, patients with baseline eGFR <60 ml/min/1.73 m^2^ were excluded (n = 960). Incident albuminuria was defined as uACR>30 mg/g in years 2 and 5, year 5 only, or year 2 when year 5 data was missing.

## Results

This substudy of ONTARGET and TRANSCEND included 3128 patients from ONTARGET and 595 patients from TRANSCEND. These patients were mostly male patients (Table 1) with either prevalent (n = 2920) or incident (n = 803) diabetes. Most of the patients (75%) in this substudy were self-described Europeans. The baseline characteristics of the patients who were included in this genetic substudy of diabetic patients from ONTARGET and TRANSCEND were in general similar to the baseline characteristics of all diabetic patients in ONTARGET and TRANSCEND (Table 1). However, the fraction of patients in the European, Asian, and Native Latin self- reported ethnicities was different in study participants compared with all diabetics in ONTARGET and TRANSCEND, likely reflecting the low availability of DNA from patients recruited in Asia and Latin America.

**Figure 1:**
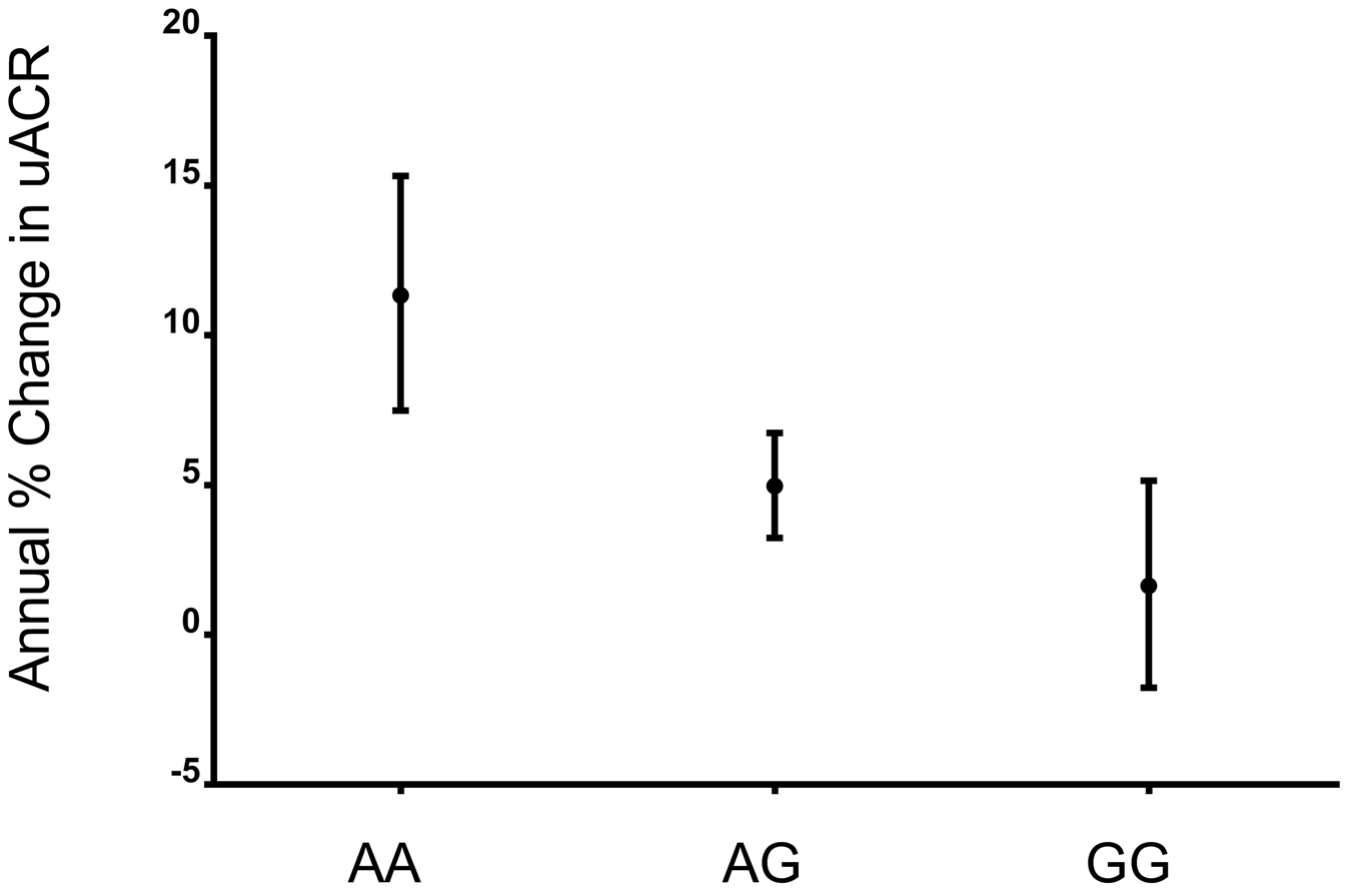
Whiskers denote 95% confidence intervals derived from linear mixed models adjusted for age, sex, and principal components of genetic variation.

In this substudy, we investigated 16 SNPs that were previously reported to be associated with eGFR in cross-sectional studies (Table 2). First we investigated the association of these SNPs with annual rate of increase in albuminuria among those with European ancestry and among all ethnic groups (Table 3). We found that 1 of the 16 SNPs (rs267734 in CERS2) was associated with differential annual rate of increase in albuminuria after accounting for testing 16 SNPs (P = 0.0015, Bonferroni corrected P value for α = 0.05 of 16 tests is 0.0032) and after adjustment for age, sex, ethnicity, and principal components of genetic variability. The median annual rate of increase in albuminuria in this population, regardless of genotype, was 6.8% (IQR 2.1% to 12.9%). However, among carriers of 2 risk alleles of this SNP, albuminuria increased at an annual rate of 11.3% (95%CI, 7.5% to 15.3%), compared with an annual rate of 5.0% (95%CI; 3.3% to 6.8%) for carriers of one risk allele (heterozygotes), and 1.7% (95%CI, −1.7% to 5.3%) for carriers of no risk alleles (Figure 1). Further adjusting this association for baseline hypertension, eGFR, uACR, smoking, study (ONTARGET or TRANSCEND), and treatment group did not appreciably change the association of rs267734 with rate of increase in albuminuria (P = 0.0034). Among patients who were normo-albuminuric at baseline (uACR<30 mg/g), each risk allele was associated with 50% increased risk of incident albuminuria (OR = 1.50; 95%CI 1.15 to 1.95; P = 0.003) after adjusting for age, sex, ethnicity, principal components of genetic variability, baseline hypertension, eGFR, uACR, smoking, study (ONTARGET or TRANSCEND), and treatment group.

**Table 2:** SNPs associated with eGFR in cross-sectional studies.

SNP	Lead SNP	LD (r^2^)	Alleles (Risk/Non-risk)	Allele Frequency (Risk)	Locus	Genes
rs267734			A/G	0.79	1q21.3	CERS2, ANXA9
rs1260326			G/A	0.59	2p23.3	GCKR
rs13538			A/G	0.78	2p13.1	NAT8
rs347685			A/C	0.73	3q23	TFDP2
rs17319721			A/G	0.42	4q21.1	SHROOM3
rs11959928			A/T	0.45	5p13.1	DAB2
rs6420094			C/T	0.33	5q35.3	SLC34A1
rs881858			A/G	0.70	6p21.1	VEGFA
rs7805747			A/G	0.28	7q36.1	PRKAG2
rs17786744	rs10109414	1	G/A	0.42	8p21.2	STC1
rs4744712			A/C	0.39	9q21.11	PIP5K1B
rs653178			G/A	0.50	12q24.12	ATXN2
rs626277			A/C	0.61	13q21.33	DACH1
rs1394125			A/G	0.35	15q24.2	UBE2Q2
rs4293393	rs12917707	1	A/G	0.82	16p12.3	UMOD
rs12460876			T/C	0.61	19q13.11	SLC7A9

LD, linkage disequilibrium.

**Table 3:** Association between SNPs and change in albuminuria.

	Europeans				All Ethnic Groups			
SNP	Annual change (%) per number of risk alleles				Annual change (%) per number of risk alleles			
	0	1	2	P value	0	1	2	P value
rs267734	1.99	4.99	8.07	0.0067	1.75	5.03	11.33	0.0015
rs1260326	8.61	7.07	5.56	0.11	8.14	9.04	11.68	0.44
rs13538	5.56	6.34	7.13	0.50	6.04	9.18	11.44	0.40
rs347685	5.23	6.31	7.41	0.31	9.00	10.28	9.97	0.45
rs17319721	6.73	6.77	6.80	0.97	11.27	7.05	6.72	0.63
rs11959928	6.20	6.85	7.51	0.50	10.25	9.93	8.01	0.57
rs6420094	5.92	7.24	8.58	0.20	9.28	11.05	10.53	0.15
rs881858	8.32	7.22	6.14	0.30	13.33	11.21	8.33	0.086
rs7805747	6.41	7.07	7.74	0.52	10.74	7.35	7.69	0.89
rs17786744	5.81	6.94	8.09	0.23	9.46	10.38	8.81	0.23
rs4744712	6.50	6.85	7.20	0.72	8.80	11.15	12.27	0.42
rs653178	7.35	6.77	6.20	0.54	10.45	7.68	6.45	0.49
rs626277	8.38	7.07	5.78	0.18	10.33	8.65	8.96	0.75
rs1394125	6.06	7.08	8.12	0.30	10.83	9.01	8.22	0.54
rs4293393	4.81	6.00	7.21	0.33	6.05	9.06	10.19	0.37
rs12460876	5.58	6.57	7.58	0.30	9.64	9.96	8.95	0.40

Results were adjusted for age, sex, 10 largest principal components of genetic variation and self-reported ethnicity (for “all ethnic groups” results).

**Figure 2:**
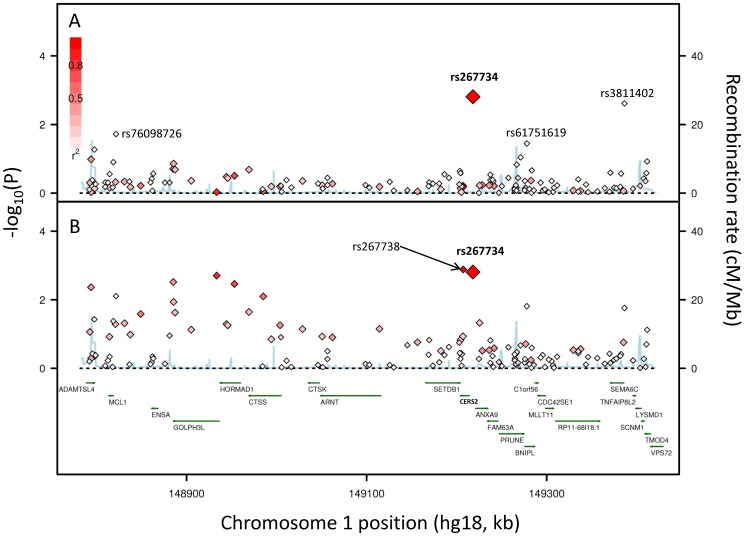
Regional plot for the CERS2 locus. SNPs are plotted by association P value of linear mixed models adjusted for age, sex, and principal components of genetic variation for the association between SNP and annual rate of change in albuminuria. and genomic position (NCBI Build 36). The original hit (rs267734) is labeled. The magnitude of linkage disequilibrium (r^2^) between each SNP and rs267734 is indicated by the intensity of the red coloring. Estimates of recombination rates are shown by the blue line. Gene positions are indicated by green arrows. Gene names are labeled. Linkage disequilibrium and recombination rates were estimated from the Utah residents of Northern and Western European ancestry (CEU) HapMap population (release 22). Plots were prepared using SNAP [22]. Panel A: P values adjusted for rs267734. Panel B: P values not adjusted for rs267734.

We investigated whether other SNPs in the rs267734 locus were also associated with annual rate of increase in albuminuria (Figure 2). An investigation of 144 SNPs in this locus in the combined ethnic groups found that 19 of these SNPs were nominally associated with annual rate of increase in albuminuria (P<0.05, Table S1). However, after adjusting the associations for the original CERS2 SNP (rs267734) only 3 SNPs remained associated with albuminuria progression (rs3811402, P = 0.0024; rs61751619, P = 0.036; and rs76098726, P = 0.019). These 3 SNPs were in low linkage disequilibrium with the original CERS2 SNP (r^2^<0.02 in Europeans). Of the 19 SNPs that were associated with annual rate of increase prior to adjusting for rs267734, only one SNP in this locus (rs267738) had an association P value smaller than rs267734 (P = 0.0013). This SNP (rs267738) encodes a glutamic acid to alanine change at position 115 of the protein encoded by CERS2. These two SNPs in CERS2 (rs267734 and rs267738) are highly correlated (linkage disequilibrium r^2^ = 0.94) in those with European ancestry in this substudy.

We also investigated the association of these 16 SNPs with baseline albuminuria levels (Table 4) and found that one SNP (rs13538 in NAT8) was associated with albuminuria in a combined analysis of all ethnic groups (P = 0.009), although this association did not reach statistical significance when Bonferroni correction was applied to adjust for testing 16 SNPs. Paradoxically, the rs13538 allele that was reported to be associated with low eGFR was found to be associated with low baseline albuminuria in our study.

**Table 4:** Association between SNPs and baseline albuminuria.

	Europeans		All Ethnic Groups	
SNP	β	P value	β	P value
rs267734	0.0043	0.94	0.015	0.78
rs1260326	−0.0439	0.34	−0.0381	0.36
rs13538	−0.1048	0.057	−0.1337	0.009
rs347685	0.0293	0.57	0.0175	0.71
rs17319721	−0.0096	0.84	−0.0684	0.51
rs11959928	0.0183	0.69	0.0111	0.79
rs6420094	0.0131	0.79	0.018	0.69
rs881858	−0.0449	0.36	−0.0598	0.18
rs7805747	−0.0651	0.19	−0.0664	0.16
rs17786744	−0.0600	0.18	−0.0258	0.74
rs4744712	−0.0462	0.32	−0.066	0.12
rs653178	0.0152	0.74	−0.0019	0.97
rs626277	−0.0069	0.88	−0.0009	0.98
rs1394125	−0.0090	0.85	0.0072	0.87
rs4293393	−0.0612	0.30	−0.0623	0.25
rs12460876	0.0021	0.96	0.0045	0.93

β is the expected change in log-transformed baseline albuminuria for each risk allele. Models are adjusted for age, sex, 10 largest principal components of genetic variation, and self-reported ethnicity.

Finally, we sought to confirm the previously reported association of these 16 SNPs with baseline eGFR in this study. Among patients with self-described European ancestry—the largest ethnic group in this substudy—we found that 3 of the 16 SNPs (rs267734 in CERS2, rs347685 in TFDP2, and rs653178 in ATXN2) were associated with baseline eGFR (P≤0.05, Table 5). As expected, patients carrying the pre-specified risk alleles of 13 of the 16 SNPs had lower eGFR than non-carriers. A combined analysis of all ethnic groups found that only one SNP (rs347685 in TFDP2) was associated with eGFR (P = 0.049) and that carriers of the risk alleles of 10 of the 16 SNPs had lower eGFR than non-carriers. None of the SNPs were associated with annual rate of eGFR change among either Europeans or in a combined analysis of all ethnic groups (Table 6).

**Table 5:** Association between SNPs and baseline eGFR.

	Europeans		All Ethnic Groups	
SNP	β	P value	β	P value
rs267734	−0.0269	0.002	0.0038	0.89
rs1260326	−0.0087	0.23	−0.0195	0.33
rs13538	−0.004	0.65	0.0178	0.34
rs347685	−0.016	0.05	−0.0147	0.049
rs17319721	0.006	0.41	0.013	0.39
rs11959928	−0.0097	0.18	−0.0088	0.30
rs6420094	−0.0048	0.53	−0.0434	0.064
rs881858	−0.0147	0.062	0.0164	0.44
rs7805747	−0.013	0.10	−0.0098	0.19
rs17786744	−0.009	0.21	−0.0099	0.13
rs4744712	−0.0065	0.38	−0.0062	0.35
rs653178	−0.0144	0.046	−0.013	0.06
rs626277	0.0051	0.49	0.0044	0.52
rs1394125	−0.0056	0.45	−0.0063	0.36
rs4293393	−0.0175	0.063	−0.0172	0.047
rs12460876	0.0045	0.54	0.0028	0.67

β is the expected change in log-transformed baseline eGFR for each risk allele. Models are adjusted for age, sex, 10 largest principal components of genetic variation, and self-reported ethnicity.

**Table 6:** Association between SNPs and change in eGFR

	Europeans				All Ethnic Groups*			
SNP	Annual change (%) by number of risk alleles				Annual change (%) by number of risk alleles			
	0	1	2	P value	0	1	2	P value
rs267734	−2.53	−2.45	−2.38	0.70	−2.52	−2.45	−2.69	0.75
rs1260326	−2.37	−2.40	−2.44	0.83	−2.44	−2.73	−2.59	0.82
rs13538	−2.54	−2.46	−2.38	0.72	−2.52	−2.48	−2.70	0.80
rs347685	−2.25	−2.37	−2.49	0.54	−2.36	−2.56	−2.63	0.48
rs17319721	−2.23	−2.45	−2.66	0.22	−2.62	−2.46	−2.67	0.22
rs11959928	−2.47	−2.41	−2.35	0.71	−2.48	−3.02	−3.66	0.28
rs6420094	−2.50	−2.37	−2.25	0.50	−2.76	−2.4	−2.26	0.40
rs881858	−2.42	−2.42	−2.42	1.00	−2.44	−2.46	−2.74	0.85
rs7805747	−2.39	−2.44	−2.49	0.78	−2.61	−2.47	−2.55	0.66
rs17786744	−2.41	−2.42	−2.42	0.97	−2.49	−2.64	−2.49	0.89
rs4744712	−2.40	−2.41	−2.43	0.92	−3.05	−2.56	−2.38	0.45
rs653178	−2.50	−2.41	−2.32	0.60	−2.66	−2.89	−2.89	0.74
rs626277	−2.63	−2.45	−2.28	0.31	−2.72	−2.58	−2.32	0.40
rs1394125	−2.47	−2.40	−2.32	0.68	−2.74	−2.41	−2.33	0.60
rs4293393	−2.28	−2.36	−2.44	0.71	−2.35	−2.42	−2.66	0.73
rs12460876	−2.31	−2.40	−2.48	0.62	−2.41	−2.63	−2.55	0.51

Results were adjusted for age, sex, 10 largest principal components of genetic variation and self-reported ethnicity (for “all ethnic groups” results)

*Mixed regression models did not converge for the self-reported Native Latin group and they were excluded from the analysis.

## Discussion

In a pre-specified investigation of 16 SNPs that were previously found to be associated with eGFR we found that one SNP (rs267734) in the CERS2 gene was associated with annual rate of increase in albuminuria in patients with diabetes from ONTARGET and TRANSCEND after adjusting for other established risk factors for CKD progression including baseline albuminuria and baseline eGFR. Each risk allele of rs267734 was associated with 50% increased risk of incident albuminuria.

This SNP was not associated with baseline albuminuria in our study. It was also not reported to be associated with baseline albuminuria in large cross sectional analysis of population-based studies [8], [20]. Thus, this SNP may only be associated with rate of increase in albuminuria in this population of patients with diabetes who were predisposed to a rapid change in albuminuria. The rs267734 SNP was not associated with deterioration of eGFR—another indicator of worsening kidney function. That this SNP is associated with rate of increase in albuminuria and not with rate of deterioration of eGFR may suggest that increase in albuminuria is more rapid, or that increase in albuminuria precedes deterioration of eGFR among patients with diabetes.

CERS2 encodes ceramide synthase 2, one of 6 mammalian ceramide synthases which acylate dihydro-sphingosine to form dihydro-ceramide or sphingosine to form ceramide [21]. Ceramides are intra- and extra-cellular signaling molecules that play a role in several pathological and physiological processes including inflammation, diabetes, and angiogenesis [21]. CERS2 mRNA has been reported to be the most abundantly expressed ceramide synthase and to be highly expressed in both liver and kidney [22]. One study of CERS2 knock-out mice reported morphological changes in the kidney [23], another study reported normal kidney morphology and function [24]. The robust expression of CERS2 in mouse kidney could indicate that it might play a role in kidney physiology. However, this role might be obscured by other physiological processes in normal mice. We investigated other SNPs in the CERS2 locus and found another SNP in the CERS2 gene (rs267738) that was as strongly associated with increase in albuminuria as rs267734. These two SNPs are in strong linkage disequilibrium among Europeans; however rs267738 could conceivably be more likely to explain the association because it encodes an amino acid change (glutamic acid to alanine) at position 115 of the CERS2 encoded protein, however, we have no functional data to indicate whether this amino acid change is associated with change in ceramide synthase activity or stability. The more common allele (glutamic acid, 80% frequency among those with European ancestry) is associated with increased rate of change in albuminuria. It would be interesting to investigate the effect of the glutamic acid to alanine substitution at this position on CERS2 activity because it could indicate whether inhibition of ceramide synthase 2 activity would inhibit or promote worsening of albuminuria.

This study has several limitations. Albuminuria was assessed from spot urine at baseline, year 2, and year 5 of follow-up. It is likely that a more accurate assessment of albuminuria and albuminuria progression would be achieved from multiple measurements on consecutive days, and from more frequent longitudinal sampling. We limited the patients in this substudy to those with prevalent or incident diabetes. However, we have no information on the duration of diabetes among those with prevalent diabetes, and thus were unable to adjust the association for potential effects of diabetes duration on albuminuria progression.

In conclusion, we found that a SNP in the CERS2 gene that was previously found to be associated with eGFR was also associated with increase in albuminuria among patients with diabetes and elevated cardiovascular risk. Although this observation has reasonable biological plausibility, it requires replication in additional studies.

## Supporting Information

Table S1 Association between SNPs in the CERS2 locus and change in albuminuria.
(DOC)
