# Supplementary material for: A Gene Variant in CERS2 Is Associated with Rate of Increase in Albuminuria in Patients with Diabetes from ONTARGET and TRANSCEND
Source: PLoS One. 2014 Sep 19;9(9):e106631. doi: 10.1371/journal.pone.0106631 (PMC4169514; doi:10.1371/journal.pone.0106631)
Supplement: Table S1 — Association between SNPs in the CERS2 locus and change in albuminuria. (DOC) [file pone.0106631.s001.doc]

**Table S1.** Association between SNPs in the CERS2 locus and change in albuminuria

|  |  |  |  | **Not adjusted for rs267734** | | | |  | **Adjusted for rs267734** | | | |
| --- | --- | --- | --- | --- | --- | --- | --- | --- | --- | --- | --- | --- |
|  |  |  |  | **Annual change (%)** | | | **P value** |  | **Annual change (%)** | | | **P value** |
|  | **Allele** | |  | **by number of alleles** | | |  |  | **by number of alleles** | | |  |
| SNP | Reference | Non-reference |  | 0 | 1 | 2 |  |  | 0 | 1 | 2 |  |
| rs41317513 | A | G |  | 8.95 | 7.88 | 10.24 | 0.63 |  | 9.35 | 8.77 | 11.43 | 0.79 |
| rs41317515 | G | C |  | 9.35 | 9.99 | 10.28 | 0.087 |  | 9.62 | 11.07 | 12.06 | 0.50 |
| rs12124948 | T | C |  | 6.87 | 9.39 | 11.23 | 0.0043 |  | 8.88 | 10.56 | 11.47 | 0.10 |
| rs9659073 | A | G |  | 10.80 | 10.18 | 9.06 | 0.51 |  | 12.80 | 11.42 | 9.33 | 0.93 |
| rs12119250 | A | G |  | 17.82 | 12.93 | 9.83 | 0.37 |  | 18.86 | 14.14 | 10.96 | 0.42 |
| rs10888382 | T | C |  | 17.10 | 13.25 | 8.80 | 0.037 |  | 17.77 | 14.19 | 9.75 | 0.054 |
| rs11204664 | T | C |  | 11.22 | 10.19 | 9.17 | 0.44 |  | 11.34 | 10.98 | 10.79 | 0.73 |
| rs56411234 | A | G |  | 11.34 | 9.06 | 10.24 | 0.42 |  | 11.55 | 9.83 | 11.34 | 0.55 |
| rs80275252 | T | C |  | 8.75 | 10.15 | 10.21 | 0.84 |  | 8.52 | 10.67 | 11.39 | 0.66 |
| rs11204666 | A | G |  | 8.12 | 10.10 | 10.10 | 0.85 |  | 8.43 | 10.84 | 11.46 | 0.49 |
| rs72700831 | C | T |  | 2.57 | 4.74 | 8.28 | 0.59 |  | 2.70 | 5.47 | 9.57 | 0.50 |
| rs878471 | G | A |  | 9.54 | 10.23 | 8.98 | 0.12 |  | 9.71 | 11.49 | 10.88 | 0.69 |
| rs35392872 | A | G |  | 4.60 | 5.89 | 10.28 | 0.46 |  | 4.77 | 6.68 | 11.41 | 0.28 |
| rs35661734 | C | T |  | 7.23 | 7.47 | 10.32 | 0.92 |  | 7.23 | 8.16 | 11.54 | 0.69 |
| rs16837903 | T | C |  | 11.67 | 11.18 | 9.75 | 0.042 |  | 12.19 | 12.49 | 10.83 | 0.12 |
| rs12125672 | A | T |  | 10.97 | 10.02 | 7.25 | 0.051 |  | 11.13 | 11.28 | 8.64 | 0.47 |
| rs76098726 | C | T |  | 16.19 | 11.17 | 9.96 | 0.0079 |  | 16.40 | 11.90 | 11.02 | 0.019 |
| rs6698495 | T | C |  | 11.09 | 9.99 | 6.91 | 0.049 |  | 11.25 | 11.27 | 8.30 | 0.46 |
| rs11204674 | C | T |  | 7.12 | 8.59 | 10.32 | 0.77 |  | 7.30 | 9.31 | 11.54 | 0.46 |
| rs11204675 | T | G |  | 10.44 | 10.15 | 7.91 | 0.10 |  | 10.66 | 11.42 | 9.55 | 0.67 |
| rs1355637 | T | C |  | 2.90 | 5.40 | 10.80 | 0.026 |  | 7.02 | 7.77 | 11.27 | 0.61 |
| rs77604525 | G | T |  | 6.34 | 6.77 | 10.28 | 0.76 |  | 6.65 | 7.68 | 11.48 | 0.49 |
| rs1053701 | T | C |  | 6.75 | 8.27 | 10.42 | 0.67 |  | 6.88 | 9.03 | 11.64 | 0.38 |
| rs11204682 | A | C |  | 9.10 | 10.32 | 9.90 | 0.46 |  | 9.39 | 11.02 | 11.21 | 0.85 |
| rs7517 | A | C |  | 6.68 | 8.79 | 10.35 | 0.52 |  | 6.87 | 9.55 | 11.63 | 0.27 |
| rs116173394 | A | G |  | 5.67 | 6.36 | 8.30 | 0.73 |  | 5.67 | 7.08 | 9.83 | 0.49 |
| rs12067556 | A | G |  | 9.82 | 10.22 | 9.46 | 0.11 |  | 9.96 | 11.13 | 10.89 | 0.49 |
| rs2055975 | A | C |  | 5.42 | 8.45 | 11.17 | 0.0030 |  | 8.05 | 10.06 | 11.35 | 0.20 |
| rs9733 | T | G |  | 11.35 | 10.88 | 8.21 | 0.012 |  | 11.76 | 12.07 | 9.61 | 0.14 |
| rs3087960 | G | C |  | 10.50 | 10.21 | 8.64 | 0.024 |  | 10.63 | 11.11 | 9.95 | 0.21 |
| rs10788792 | C | A |  | 9.78 | 10.28 | 9.37 | 0.075 |  | 9.91 | 11.22 | 10.76 | 0.44 |
| rs41305070 | G | A |  | 1.92 | 6.50 | 10.78 | 0.0020 |  | 11.46 | 11.38 | 10.98 | 0.93 |
| rs11807450 | A | G |  | 9.95 | 10.45 | 9.03 | 0.051 |  | 10.08 | 11.39 | 10.37 | 0.33 |
| rs1336900 | A | G |  | 9.98 | 10.48 | 9.06 | 0.055 |  | 10.12 | 11.44 | 10.39 | 0.36 |
| rs34593358 | T | A |  | 3.39 | 8.45 | 10.80 | 0.0034 |  | 10.43 | 11.34 | 10.98 | 0.31 |
| rs1136808 | C | G |  | 11.21 | 10.43 | 8.90 | 0.023 |  | 11.36 | 11.30 | 10.20 | 0.21 |
| rs72704612 | G | A |  | 7.81 | 7.55 | 10.16 | 0.87 |  | 7.82 | 8.28 | 11.35 | 0.77 |
| rs75056606 | G | T |  | 2.49 | 7.45 | 10.35 | 0.0080 |  | 13.00 | 12.58 | 10.95 | 0.88 |
| rs10888390 | A | G |  | 10.18 | 10.10 | 9.70 | 0.14 |  | 10.29 | 11.04 | 11.06 | 0.64 |
| rs41271951 | G | A |  | 0.99 | 4.20 | 10.22 | 0.055 |  | 19.44 | 16.43 | 11.30 | 0.64 |
| rs1136774 | C | T |  | 10.07 | 10.28 | 8.54 | 0.12 |  | 10.29 | 11.79 | 11.08 | 0.87 |
| rs3754212 | G | A |  | 10.40 | 9.99 | 9.46 | 0.12 |  | 10.47 | 10.93 | 10.85 | 0.61 |
| rs34711732 | T | C |  | 8.68 | 8.44 | 10.02 | 0.93 |  | 9.58 | 9.34 | 11.15 | 0.91 |
| rs75999286 | T | C |  | 4.16 | 5.56 | 8.33 | 0.60 |  | 3.96 | 6.15 | 9.83 | 0.42 |
| rs76580510 | G | C |  | 6.32 | 6.62 | 8.31 | 0.91 |  | 6.13 | 7.21 | 9.80 | 0.68 |
| rs34481938 | G | C |  | 7.87 | 7.46 | 10.19 | 0.88 |  | 8.17 | 8.39 | 11.32 | 0.97 |
| rs10888395 | A | G |  | 11.08 | 10.08 | 9.11 | 0.072 |  | 11.20 | 10.99 | 10.43 | 0.44 |
| rs188415602 | C | T |  | -7.95 | 1.44 | 8.97 | 0.28 |  | -1.74 | 5.26 | 9.76 | 0.46 |
| rs12746973 | A | G |  | 7.16 | 7.56 | 10.18 | 0.80 |  | 7.27 | 8.32 | 11.36 | 0.52 |
| rs11552229 | G | A |  | 10.41 | 9.99 | 9.49 | 0.12 |  | 10.48 | 10.92 | 10.89 | 0.59 |
| rs10305748 | T | C |  | 6.65 | 6.92 | 8.61 | 0.93 |  | 6.59 | 7.59 | 10.03 | 0.74 |
| rs10305741 | C | A |  | 24.56 | 15.93 | 7.80 | 0.54 |  | 24.10 | 16.46 | 8.84 | 0.58 |
| rs10305740 | A | G |  | 32.35 | 20.23 | 9.89 | 0.32 |  | 31.53 | 20.54 | 10.93 | 0.36 |
| rs10305724 | A | G |  | 1.58 | 4.46 | 10.21 | 0.12 |  | 21.41 | 17.35 | 11.35 | 0.53 |
| rs10305667 | T | C |  | 9.95 | 10.07 | 9.82 | 0.75 |  | 9.89 | 10.86 | 11.23 | 0.58 |
| rs12406660 | C | T |  | 9.39 | 10.44 | 9.59 | 0.79 |  | 9.39 | 11.14 | 10.93 | 0.69 |
| rs11204737 | C | T |  | 10.42 | 10.10 | 7.72 | 0.071 |  | 10.60 | 11.50 | 9.93 | 0.65 |
| rs10305649 | A | C |  | 5.29 | 6.21 | 8.79 | 0.70 |  | 5.34 | 6.93 | 10.13 | 0.50 |
| rs75067938 | C | G |  | 6.54 | 6.90 | 10.12 | 0.80 |  | 6.65 | 7.74 | 11.34 | 0.47 |
| rs116275309 | A | G |  | 7.45 | 7.27 | 10.24 | 0.94 |  | 7.47 | 7.97 | 11.45 | 0.85 |
| rs115298177 | T | C |  | 1.04 | 4.10 | 10.10 | 0.21 |  | 7.19 | 9.72 | 11.14 | 0.83 |
| rs12126004 | T | C |  | 10.75 | 10.07 | 6.63 | 0.17 |  | 10.87 | 11.62 | 9.28 | 0.89 |
| rs17661062 | A | T |  | 10.98 | 11.50 | 10.08 | 0.40 |  | 12.26 | 12.53 | 11.23 | 0.63 |
| rs11204744 | G | A |  | 10.13 | 11.22 | 9.40 | 0.15 |  | 10.20 | 11.81 | 10.70 | 0.49 |
| rs34207591 | G | A |  | 7.14 | 7.48 | 10.23 | 0.83 |  | 7.17 | 8.19 | 11.40 | 0.53 |
| rs41310889 | T | C |  | 4.28 | 5.57 | 10.05 | 0.67 |  | 4.32 | 6.25 | 11.03 | 0.52 |
| rs7528773 | A | G |  | 10.54 | 9.90 | 9.68 | 0.31 |  | 10.63 | 10.82 | 11.19 | 0.78 |
| rs6694978 | T | C |  | 14.65 | 10.59 | 10.18 | 0.41 |  | 14.64 | 11.20 | 11.28 | 0.28 |
| rs79228431 | A | G |  | 12.36 | 9.52 | 10.25 | 0.24 |  | 12.50 | 10.24 | 11.36 | 0.36 |
| rs74681459 | G | C |  | -2.84 | 3.50 | 10.66 | 0.18 |  | 3.04 | 6.81 | 11.39 | 0.55 |
| rs1045628 | C | T |  | 3.00 | 5.05 | 8.68 | 0.36 |  | 3.08 | 5.79 | 10.06 | 0.23 |
| rs1546376 | G | A |  | 10.59 | 9.95 | 9.54 | 0.15 |  | 10.66 | 10.85 | 11.07 | 0.72 |
| rs72706512 | G | A |  | 6.27 | 7.91 | 10.40 | 0.53 |  | 6.29 | 8.59 | 11.65 | 0.26 |
| rs8444 | A | G |  | 10.51 | 9.89 | 9.71 | 0.38 |  | 10.56 | 10.79 | 11.30 | 0.93 |
| rs41308401 | G | A |  | 5.99 | 6.47 | 8.72 | 0.84 |  | 6.08 | 7.20 | 10.07 | 0.63 |
| rs59988025 | T | C |  | 10.70 | 10.12 | 9.16 | 0.12 |  | 10.79 | 11.00 | 10.57 | 0.61 |
| rs267738 | C | A |  | 1.61 | 4.97 | 11.31 | 0.0013 |  | 3.26 | 6.51 | 11.47 | 0.65 |
| rs267734 | G | A |  | 1.75 | 5.03 | 11.33 | 0.0015 |  | NA | NA | NA | NA |
| rs11204752 | T | C |  | 9.06 | 10.45 | 10.18 | 0.53 |  | 10.78 | 11.67 | 10.81 | 0.99 |
| rs267733 | G | A |  | 1.83 | 4.87 | 11.44 | 0.047 |  | 6.53 | 7.44 | 11.61 | 0.60 |
| rs12033744 | A | G |  | 11.94 | 10.70 | 9.63 | 0.15 |  | 11.89 | 11.08 | 10.99 | 0.36 |
| rs11204755 | G | T |  | 3.97 | 6.70 | 10.18 | 0.31 |  | 14.47 | 12.89 | 11.30 | 0.58 |
| rs4590677 | T | C |  | 11.08 | 8.70 | 5.17 | 0.021 |  | 11.59 | 10.16 | 6.94 | 0.10 |
| rs75172050 | T | C |  | 3.89 | 6.57 | 10.20 | 0.29 |  | 14.38 | 12.84 | 11.32 | 0.59 |
| rs74602660 | C | G |  | 7.38 | 9.06 | 10.00 | 0.69 |  | 7.55 | 9.89 | 11.27 | 0.41 |
| rs1673160 | T | A |  | 11.12 | 10.14 | 6.62 | 0.15 |  | 11.79 | 11.36 | 8.17 | 0.29 |
| rs78915130 | G | C |  | 0.21 | 5.49 | 10.50 | 0.44 |  | 3.98 | 6.47 | 11.52 | 0.41 |
| rs771204 | G | A |  | 7.72 | 9.61 | 10.07 | 0.91 |  | 7.80 | 10.43 | 11.41 | 0.45 |
| rs41310885 | A | T |  | 3.64 | 6.65 | 10.27 | 0.26 |  | 14.35 | 12.86 | 11.39 | 0.62 |
| rs7548516 | C | T |  | 11.04 | 9.92 | 9.99 | 0.66 |  | 11.08 | 10.74 | 11.50 | 0.98 |
| rs12119409 | C | G |  | 10.47 | 9.78 | 8.36 | 0.86 |  | 11.19 | 10.97 | 9.46 | 0.63 |
| rs3738480 | A | G |  | 7.38 | 9.45 | 10.03 | 0.24 |  | 8.84 | 10.68 | 11.00 | 0.36 |
| rs3820539 | A | G |  | 9.97 | 10.46 | 10.22 | 0.39 |  | 10.21 | 11.29 | 11.68 | 0.77 |
| rs3738479 | A | T |  | 11.14 | 10.50 | 9.28 | 0.59 |  | 11.49 | 11.46 | 10.85 | 0.85 |
| rs3738478 | A | G |  | 10.46 | 10.26 | 8.84 | 0.52 |  | 10.85 | 11.38 | 10.70 | 0.70 |
| rs6673178 | A | T |  | 9.55 | 10.54 | 9.24 | 0.75 |  | 10.27 | 11.78 | 10.33 | 0.59 |
| rs11204762 | G | A |  | 9.88 | 10.48 | 9.83 | 0.36 |  | 10.28 | 11.37 | 11.23 | 0.70 |
| rs55717234 | A | G |  | 11.05 | 10.11 | 7.57 | 0.82 |  | 12.00 | 11.22 | 8.81 | 0.85 |
| rs35550545 | A | G |  | 5.37 | 6.60 | 10.50 | 0.34 |  | 5.61 | 7.51 | 11.76 | 0.15 |
| rs6691701 | G | A |  | 9.91 | 9.38 | 9.72 | 0.68 |  | 11.28 | 10.87 | 10.64 | 0.36 |
| rs7532312 | C | A |  | 4.69 | 6.73 | 10.77 | 0.21 |  | 4.92 | 7.27 | 12.05 | 0.091 |
| rs72706550 | T | C |  | 8.21 | 7.58 | 10.29 | 0.83 |  | 8.17 | 8.23 | 11.39 | 0.98 |
| rs12135409 | T | C |  | 7.83 | 7.31 | 10.48 | 0.68 |  | 8.78 | 8.52 | 11.55 | 0.83 |
| rs41266605 | G | A |  | 4.43 | 6.03 | 10.45 | 0.54 |  | 4.21 | 6.78 | 11.67 | 0.44 |
| rs12070288 | G | C |  | 5.68 | 8.27 | 10.10 | 0.19 |  | 13.36 | 12.34 | 11.13 | 0.73 |
| rs61751619 | T | C |  | 12.94 | 9.51 | 10.00 | 0.015 |  | 13.43 | 10.41 | 11.12 | 0.036 |
| rs6694158 | C | G |  | 7.95 | 8.78 | 10.32 | 0.75 |  | 9.25 | 9.72 | 11.37 | 0.76 |
| rs12068365 | A | G |  | 9.47 | 9.85 | 9.95 | 0.58 |  | 17.56 | 14.28 | 11.01 | 0.43 |
| rs11204764 | C | T |  | 8.95 | 9.29 | 9.71 | 0.96 |  | 10.46 | 10.23 | 10.84 | 0.97 |
| rs76483658 | G | A |  | 0.75 | 3.95 | 10.58 | 0.25 |  | 1.03 | 4.78 | 11.85 | 0.22 |
| rs6587553 | A | G |  | 7.11 | 8.48 | 10.45 | 0.80 |  | 8.48 | 9.41 | 11.50 | 0.83 |
| rs77503411 | A | G |  | 32.22 | 20.43 | 10.21 | 0.48 |  | 32.75 | 21.39 | 11.34 | 0.50 |
| rs2864700 | C | T |  | 6.87 | 9.64 | 10.95 | 0.41 |  | 9.33 | 11.06 | 11.51 | 0.85 |
| rs75309199 | G | C |  | 6.16 | 6.47 | 6.77 | 0.92 |  | 6.43 | 7.27 | 8.12 | 0.78 |
| rs6694357 | C | A |  | 5.25 | 6.24 | 10.63 | 0.55 |  | 8.66 | 8.54 | 11.65 | 0.95 |
| rs6674232 | C | T |  | 6.01 | 6.91 | 10.51 | 0.62 |  | 6.36 | 7.76 | 11.72 | 0.45 |
| rs15740 | G | A |  | 7.52 | 8.02 | 11.14 | 0.81 |  | 8.98 | 9.13 | 12.15 | 0.93 |
| rs76902030 | T | G |  | 7.17 | 7.09 | 10.02 | 0.96 |  | 7.27 | 7.91 | 11.18 | 0.70 |
| rs12039048 | A | G |  | 11.29 | 12.39 | 10.09 | 0.86 |  | 11.22 | 12.71 | 11.05 | 0.80 |
| rs116537710 | T | C |  | 10.10 | 8.43 | 10.32 | 0.46 |  | 10.55 | 9.32 | 11.42 | 0.60 |
| rs11204774 | A | G |  | 4.22 | 8.09 | 9.92 | 0.29 |  | 9.54 | 11.36 | 10.95 | 0.83 |
| rs72706600 | C | A |  | 12.19 | 9.46 | 10.20 | 0.32 |  | 12.75 | 10.40 | 11.31 | 0.40 |
| rs4970941 | A | G |  | 6.61 | 8.85 | 10.78 | 0.36 |  | 8.90 | 10.11 | 11.49 | 0.90 |
| rs7537292 | G | C |  | 5.98 | 8.52 | 9.92 | 0.27 |  | 12.65 | 12.20 | 10.97 | 0.86 |
| rs74729159 | G | A |  | 5.86 | 6.55 | 10.27 | 0.77 |  | 5.88 | 7.26 | 11.46 | 0.56 |
| rs6676190 | G | A |  | 6.89 | 8.83 | 10.69 | 0.47 |  | 9.25 | 10.11 | 11.43 | 0.93 |
| rs6656450 | A | T |  | 7.01 | 8.70 | 10.75 | 0.50 |  | 9.36 | 9.86 | 11.51 | 0.90 |
| rs115457078 | T | C |  | 15.30 | 12.40 | 7.76 | 0.73 |  | 17.72 | 14.25 | 9.02 | 0.72 |
| rs4357530 | G | A |  | 6.70 | 8.86 | 10.64 | 0.38 |  | 8.96 | 10.19 | 11.37 | 0.91 |
| rs4971004 | T | C |  | 11.04 | 10.34 | 8.26 | 0.70 |  | 11.78 | 11.54 | 10.38 | 0.26 |
| rs4971007 | T | G |  | 7.32 | 7.57 | 11.35 | 0.57 |  | 8.66 | 8.78 | 12.31 | 0.69 |
| rs72708441 | A | G |  | 9.04 | 7.96 | 9.93 | 0.52 |  | 9.49 | 8.90 | 11.06 | 0.72 |
| rs112048428 | T | C |  | 10.62 | 8.74 | 10.10 | 0.49 |  | 10.68 | 9.44 | 11.23 | 0.66 |
| rs74949844 | A | G |  | 3.85 | 5.51 | 10.39 | 0.43 |  | 6.40 | 7.38 | 11.33 | 0.66 |
| rs11807526 | C | A |  | 9.36 | 9.56 | 9.58 | 0.39 |  | 11.24 | 10.73 | 10.65 | 0.27 |
| rs12088941 | G | A |  | 3.19 | 7.55 | 10.13 | 0.18 |  | 11.06 | 11.55 | 11.17 | 0.87 |
| rs61819222 | T | C |  | 7.67 | 7.36 | 10.18 | 0.87 |  | 10.58 | 9.47 | 11.27 | 0.56 |
| rs3811402 | C | G |  | 14.81 | 10.82 | 9.96 | 0.017 |  | 18.66 | 13.41 | 10.92 | 0.0024 |
| rs2067606 | G | A |  | 6.86 | 9.11 | 10.34 | 0.59 |  | 9.61 | 10.70 | 11.21 | 0.75 |
| rs6587557 | A | G |  | 9.87 | 9.79 | 9.44 | 0.97 |  | 12.15 | 11.29 | 10.40 | 0.62 |
| rs11577302 | A | G |  | 8.55 | 7.95 | 10.29 | 0.53 |  | 10.39 | 9.38 | 11.19 | 0.37 |
| rs72708455 | T | C |  | 8.48 | 7.82 | 9.97 | 0.75 |  | 9.35 | 8.95 | 11.10 | 0.85 |
| rs6689566 | A | G |  | 12.61 | 11.13 | 9.77 | 0.91 |  | 17.30 | 14.03 | 10.88 | 0.45 |
| rs28730723 | T | C |  | 10.20 | 8.67 | 9.94 | 0.20 |  | 11.14 | 9.84 | 11.09 | 0.26 |
| rs28730724 | A | G |  | 13.29 | 9.93 | 10.24 | 0.075 |  | 13.84 | 10.86 | 11.38 | 0.12 |
